# Immunolocalisation of phosphorylated STAT3, interleukin 11 and leukaemia inhibitory factor in endometrium of women with unexplained infertility during the implantation window

**DOI:** 10.1186/1477-7827-5-44

**Published:** 2007-11-29

**Authors:** Evdokia Dimitriadis, Andrew M Sharkey, Yee Lee Tan, Lois A Salamonsen, J Robert A Sherwin

**Affiliations:** 1 Prince Henry's Institute of Medical Research, P.O. Box 5152, Clayton, VIC, 3168, Australia; 2 Department of Pathology, Tennis Court Road, Cambridge, CB2 1QP. UK; 3 Department of Obstetrics and Gynaecology, University of Cambridge, The Rosie Hospital, Cambridge, CB2 2SW, UK

## Abstract

**Background:**

Uterine receptivity and embryo implantation are critical in the establishment of pregnancy. The diagnosis of endometrial fertility requires more precise measurements of endometrial receptivity. Interleukin (IL-11) and leukemia inhibitory factor (LIF) are essential for murine implantation and signal via intracellular phosphorylation (p) of STAT3 in the endometrium. Both cytokines are present in the endometrium of women duiring the receptive window. Endometrial IL-11, IL-11 receptor alpha (IL-11Ralpha), LIF and pSTAT3 in women with primary unexplained infertility was compared to normal fertile women during the implantation window.

**Methods:**

LH timed endometrial biopsies (LH+6 to LH+10) were collected from women with unexplained infertility and normal fertility. pSTAT3, IL-11, IL-11Ralpha and LIF production was determined by immunohistochemistry. Staining intensity was determoned by two independent observers blind to the fertility status of the patient from whom the biopsy was taken. Staining intensity and heterogeneity in each of the endometrial compartments (epithelium; stroma, including decidualized stromal cells; and vasculature) was assessed. The Mann-Whitney U test was used to analyze IL-11, pSTAT3, IL-11Ralpha and LIF immunostaining intensities in the samples.

**Results:**

IL-11, IL-11Ralpha and LIF were present predominantly in glandular epithelium, whilst luminal epithelium showed patchy staining. pSTAT3 was present in both glandular epithelium and stroma. IL-11 and pSTAT3 immunostaining was significantly lower in glandular epithelium in infertile women compared to controls (P < 0.05) whilst IL-11Ralpha and LIF staining did not differ.

**Conclusion:**

This is the first demonstration of reduced endometrial pSTAT3 and IL-11 in some women with unexplained infertility. This suggests IL-11 and pSTAT3 may be involved in the secretory transformation of glandular epithelium during receptivity. Reduced IL-11 production and STAT3 phosphorylation may contribute to unexplained infertility in some women.

## Background

Embryo implantation into the uterus is a critical step in the establishment of pregnancy and failure of this process is a major cause of infertility in women [1].

Endometrium is receptive to the embryo for a short time-period, after exposure to 17-β-estradiol followed by progesterone [2]. Embryo transfer studies in women and primates have identified the phase of uterine receptivity, the 'window of implantation', between post-ovulatory days 5–10 [3] following the luteinizing hormone (LH) surge. Implantation of the embryo between post-ovulatory days 8–10 has a high chance of resulting in a successful pregnancy [4]. Unexplained infertility accounts for approximately 30% of all infertility [5,6]. Defective uterine receptivity is thought to be a primary cause of unexplained infertility. Unexplained or primary infertility is likely due to multiple defects as it has been associated with numerous molecular and cellular disturbances in the endometrium [7,8].

Interleukin-11 (IL-11) and LIF belong to the interleukin-6 (IL-6) family of cytokines whose members exhibit pleiotropy and redundancy, with many overlapping functions [9]. IL-11 binds to IL-11 receptor (IL-11 Rα) and initiates signalling whilst LIF signals by binding to the specific LIF receptor α (LIFR). Each ligand-receptor complex forms a hetero-dimer with gp130, the common transmembrane signal transducer. In the endometrium, intracellular signalling occurs via activation of the janus kinase (JAK)/signal transducers and activators of transcription (STAT) pathway [10-12]. IL-11 and LIF are part of an exclusive group of genes that are essential for implantation in mice [13]. Both endometrial IL-11 and LIF are obligatory for implantation in mice [14-16]. STAT3 also has an important role in implantation in mice [10,17]. Blockade of phosphorylated (p) STAT3 at the time of embryo attachment reduces implantation rates in mice [18,19].

Although the role of LIF in human endometrial receptivity has been previously studied the results are conflicting [8,20]. There are very few studies examining the role of IL-11 and no studies for pSTAT3 in infertility in women [21]. IL-11, LIF and pSTAT3 are expressed by endometrial, glandular and luminal epithelium in the mid-secretory phase of the menstrual cycle in women [22-25]. Similarly, mid-secretory phase luminal and glandular epithelium express gp130, LIFRα and IL-11Rα [22,24,26,27]. Immunoreactive LIF is reduced in endometrial biopsies from infertile women [28], while uterine flushings contain maximum levels of LIF protein during the mid to late secretory phase of the menstrual cycle [29-31]. In addition, LIF is reduced in uterine flushings from women with primary infertility compared to fertile controls [29-31]. Similarly, IL-11 is present in uterine fluid [32]. These studies suggest IL-11 and LIF are secreted by the uterine glandular epithelium into the uterine lumen where they could act on the blastocyst or the endometrial luminal epithelium to facilitate blastocyst attachment and implantation.

In addition, in some women with endometriosis associated infertility, IL-11 and LIF immunoreactivity are reduced in glandular epithelium suggesting that both cytokines may also contribute to the pathology of infertility of unknown cause [12]. While, IL-11 and LIF expression is spacially and temporally distinct in mice, they are both present in glandular epithelium and utilize similar signal transduction pathways at the time of implantation in women. It is important to determine whether they potentially act in a redundant manner. In a previous study, while LIF production was not different between women with unexplained infertility and normal fertile women, there was reduced secretion of soluble gp130 from endometrial explants. This indicates it is important to also examine IL-11 and LIF's signalling components together with the production of the ligands. pSTAT3 is of particular interest since it has an important role in implantation in mice. There are no studies examining the production of IL-11 and pSTAT3 in endometrium of women with primary unexplained infertility.

We hypothesized that IL-11, IL-11Rα, LIF and pSTAT3 are dysregulated in endometrium from women with unexplained infertility during the 'window of implantation'. Endometrial protein abundance for IL-11, pSTAT3, IL-11Rα and LIF was compared in women with primary unexplained infertility and normal fertile women.

## Methods

### Participants and endometrial biopsies

Endometrial biopsies were collected from women with primary unexplained infertility of more than 2 years duration (n = 10, age 25–40 yr) and fertile age matched controls (n = 9, age 25–40 yr) between days LH+ 6 to LH+10. All women had regular menstrual cycles of 26–32 days, were not using intra-uterine contraceptives and had not used hormones for at least three months prior to surgery. Women with infertility who had partners with male factor infertility were not included in the study. The women with infertility did not have ovulation or tubal patency defects and endometriosis. All infertile patients and controls had negative laparoscopies. All women biopsied in the secretory phase of the menstrual cycle, performed home urinary LH surge tests and all biopsies were histologically dated according to the Noyes criteria [33]. Written informed consent was obtained from all patients before their operations and protocols were approved by Institutional Ethical committees, namely Southern Health Human Ethical Committee and Addenbrooke's Hospital NHS Trust. Ethical approval was also obtained from Southern Health Human Ethics Committee, Melbourne, Australia.

#### Immunohistochemistry

Tissues were stained for IL-11 and IL-11Rα with mouse anti-huIL-11 (clone 5E3) and mouse anti-huIL-11Rα (clone 4D12) antibodies (generous gifts from Dr Lorraine Robb) as previously described [23]. Briefly, dewaxed, rehydrated sections were incubated with blocking solution containing 10% horse serum (Sigma Aldrich Inc. Missouri, USA), 5% human serum and Tris Buffered saline (TBS) (pH 7.4) for 30 min. Primary antibodies were applied diluted to 5 μg/ml (IL-11) and 2.5 μg/ml (IL-11Rα) in blocking solution for 18 h at 4°C. Antibody localization was detected by sequential application of biotinylated horse anti-mouse IgG (Vector Laboratories, Burlingame, CA, USA) in blocking solution, followed by an avidin-biotin complex conjugated to HRP (Vectastain ABC Elite kit; Vector Laboratories).

LIF protein was immunostained using antiserum against human LIF protein raised in rabbit (AMRAD Pharmacia Biotech, Boronia, Vic., Australia) as previously described [23]. The primary antibody (1:2000) was applied on the tissues for 12 h at RT. The slides were washed, biotinylated swine anti-rabbit IgG (DAKO, Glostrup, Denmark;) applied, followed by streptavidin-biotin-peroxidase complex (Vector Laboratories Inc., Burlingame, CA, USA) according to the manufacturer's instructions.

Tissues were stained for pSTAT3 as previously described using rabbit polyclonal antibodies raised against human peptides (R&D Systems Inc.) [12]. Briefly, sections were preincubated with 10% non-immune horse serum (Sigma-Aldrich, Sydney, Australia), 2% normal human serum (in-house) in TBS and 0.1% Tween 20. Primary antibodies were applied overnight (17 h) at 4°C diluted to 4 μg/ml in non-immune block. Detection of positive binding was performed by the sequential application of biotinylated goat anti-rabbit IgG; (Vector Laboratories) and avidin-biotin-peroxidase conjugate (Dako, Glostrup, Denmark).

In all cases the substrate used was diaminobenzidine (DAB) (Zymed, San Francisco, USA). A section from a single block of first trimester decidua was included in each staining run for quality control and an isotype-matched negative control at the same protein concentration as the primary antibody, was included for each tissue. The immunohistochemistry procedure was conducted at least twice for each tissue and each ligand/receptor.

#### Assessment of immunostaining

Immunostaining was analyzed semi-quantitatively by two independent observers blind to the fertility status of the patient from whom the biopsy was taken. Staining intensity and heterogeneity in each of the endometrial compartments (epithelium; stroma and vasculature) was assessed and allocated a score between 0 and 3 where numbers represent, 0: no stain, 1: faint, 2: moderate and 3: strong, relative to positive and negative controls.

### Statistics

Results are expressed as mean ± SEM. The Mann-Whitney U test was used to analyze IL-11, IL-11Rα and LIF immunostaining in the infertile and fertile control samples. Differences were considered statistically significant when *P *< 0.05.

## Results

### Endometrial IL-11, IL-11Rα and LIF immunostaining in women with primary infertility and fertile controls between days LH+6 to +10

**Figure 1:**
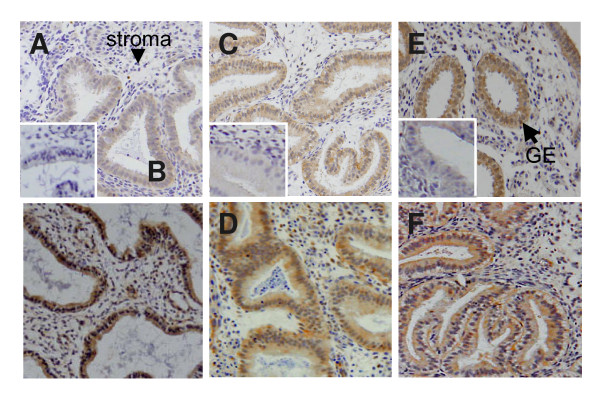
Photomicrographs representing immunostaining for IL-11, IL-11Rα and LIF of human endometrium from fertile controls and women with primary infertility. Positive staining is shown as brown pigment with blue nuclear hematoxylin counterstain. Magnification ×200. (A-B) IL-11 staining. (A) Day LH+6 tissue from woman with infertility. (B) Day LH+6 tissue from fertile woman. (C-D) IL-11Rα staining. (C) Day LH+8 tissue from woman with infertility. (D) Day LH+8 tissue from control fertile woman. (E-F) LIF staining. (E) Day LH+8 tissue from fertile woman. (F) Day LH+8 tissue from infertile woman.

**Figure 2:**
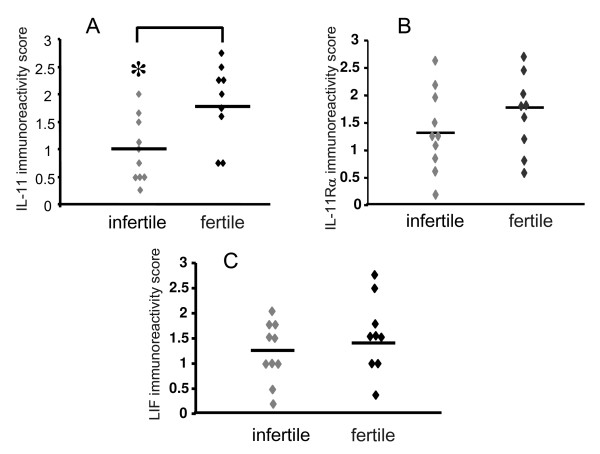
Immunostaining for IL-11, IL-11Rα and LIF in endometrial glandular epithelium from normal fertile women and women with primary infertility between days LH+6- +10 of the menstrual cycle. Relative staining intensities are represented as 0 (no staining) to 3 (high). (A) IL-11 staining. (B) IL-11Rα staining (C) LIF staining. Women with primary infertility (infertile) (n = 10). Control normal fertile women (fertile) (n = 9). Individual data shown for each parameter. Bar represents the mean value. * P < 0.05 compared to fertile control.

IL-11 staining was largely confined to the glandular epithelium in the endometrial tissue (Figure 1A and 1B). IL-11 immunoreactivity was moderate to high in glandular epithelial cells from all control fertile women and some infertile women (Figure 1A and 1B and Figure 2A). However, mean IL-11 staining in glandular epithelium was consistently more intense in tissues from control women compared to the infertile women (1.7 ± 0.2 vs 1.1 ± 0.2 respectively, P < 0.05) (Figure 2A), although there was a clear overlap between the groups. Minimal staining for IL-11 was observed in the stromal compartment in tissues from fertile and infertile women (Figure 1A and 1B). Seven tissues from both infertile and control fertile women contained luminal epithelium. IL-11 staining in luminal epithelium was low-moderate and did not differ between infertile and control groups (data not shown). There was no correlation with staining intensity and women's age (data not shown).

Similarly, IL-11Rα staining in endometrial tissues was present predominantly in glandular epithelial cells (Figure 1C and 1D). While all tissues from control and infertile women stained for IL-11Rα, the intensity ranged from minimal to high and was not different between infertile and fertile women (Figure 2B). IL-11Rα staining was low in stromal and vascular cells in all tissues and did not differ between the groups (data not shown).

LIF staining was maximal in glandular epithelium and minimal in the stromal compartment in the biopsies (Figure 1E and 1F). LIF staining intensity in glandular epithelial cells was variable within the tissues from both infertile and fertile women. It ranged from low to maximal and did not differ between the two groups (Figure 2C).

### Endometrial pSTAT3 immunostaining in women with primary infertility and fertile controls between days LH+6 to +10

**Figure 3:**
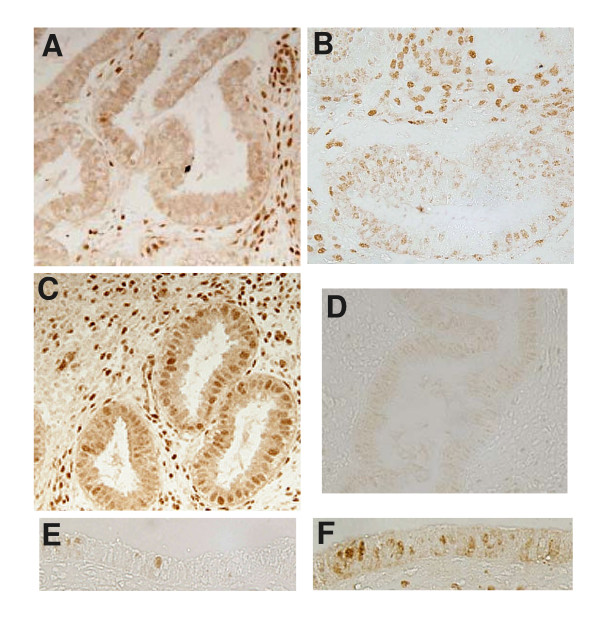
Photomicrographs representing endometrial pSTAT3 immunostaining in women with primary infertility and fertile women. Magnification ×200. (A and B) pSTAT3 immunostaining of endometrium from women with primary infertility. (A) Day LH+7 (B) Day LH+9. (C) Control fertile women day LH+6, pSTAT3 staining. (D) Day LH+10 tissue showing negative control for pSTAT3 staining. (E) Infertile women at day LH+8 showing pSTAT3 staining of luminal epithelium (F) Control fertile women at Day LH+7 showing luminal epithelium-pSTAT3.

**Figure 4:**
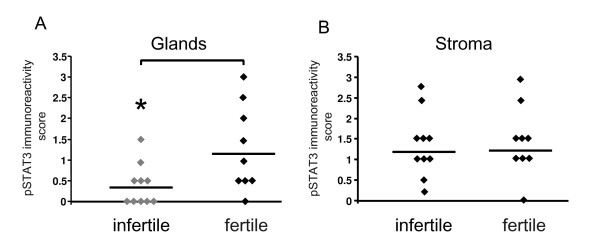
Immunostaining for pSTAT3 in endometrial glandular epithelium and stroma from normal fertile women and women with primary infertility between days LH+6- +10 of the menstrual cycle. Relative staining intensities are represented as 0 (no staining) to 3 (high). (A) Glandular epithelium. (B) Stroma. Women with primary infertility (infertile) (n = 10). Control normal fertile women (fertile) (n = 9). Individual data shown for each parameter. Bar represents the mean value. * P < 0.05 compared to fertile control.

We aimed to examine whether pSTAT3 was reduced in endometrium of women with infertility compared to normal fertile women. We also aimed to determine whether pSTAT3, IL-11's main down stream target molecule was reduced in the same women that we observed a reduction in IL-11 immunostaining. pSTAT3 staining was present in endometrial glandular epithelium and stroma (Figure 3A–C). pSTAT3 immunoreactivity ranged from absent to moderate in glandular epithelium of infertile women (Figure 3A–B and 4A). Mean pSTAT3 staining in glandular epithelium was consistently more intense in tissues from control women compared to the infertile women (1.3 ± 0.3 vs 0.4 ± 0.2 respectively, P < 0.05) (Figure 4A). No correlation was found between IL-11 and pSTAT3 staining intensity in the glandular epithelium (data not shown). Minimal to high staining for pSTAT3 was observed in the stromal compartment in tissues from fertile and infertile women and was not different between the groups of women (Figure 3A–C and 4A–B). Seven out of the ten women with infertility had very low levels of both IL-11 and pSTAT3 protein in the glandular epithelium (data no shown). Seven tissues from both infertile and control fertile women contained luminal epithelium. However, pSTAT3 staining in luminal epithelium was present in one tissue from the infertile group and two tissues from the control group (Figure 3E and 3F)

## Discussion

We demonstrated for the first time that IL-11 and pSTAT3 immunoreactivity in uterine glandular epithelium was decreased in women with unexplained infertility.

We observed a significant decrease in IL-11 and pSTAT3 in uterine glandular epithelium in some women with infertility compared to normal cycling fertile women. Unexplained infertility is likely caused by several defects therefore it is not surprising that we identified cohorts of infertile women with defective IL-11 and pSTAT3 production. IL-11 could be acting on the glandular epithelium to facilitate its secretory transformation. In addition, IL-11 has been identified in the uterine lumen of women [32]. IL-11 present in the uterine lumen could act on the endometrial uterine epithelium to facilitate attachment or adhesion of the blastocyst.

Low glandular pSTAT3 may be due to either reduced STAT3 protein production and/or factors that stimulate STAT3 phosphorylation. IL-11 and LIF are known to signal via STAT3 in endometrium [10,25]. Interestingly, glandular epithelium from women with infertility that had low IL-11 staining did not consistently have low pSTAT3 staining. LIF protein levels did not correlate with pSTAT3 levels in glandular epithelium. This suggests factors other than these cytokines regulate STAT3 protein activation. It is also plausible that IL-11 and LIF stimulate pSTAT3 in glandular epithelium but the levels of STAT3 protein may be low in glandular epithelium of some infertile women. Numerous factors including progestins stimulate pSTAT3 and STAT3 [9,34]. The regulation of pSTAT3 and STAT3 in the uterine glandular epithelium remain to be elucidated.

Minimal pSTAT3 staining was found in eight of the ten infertile women. If the levels of pSTAT3 in some women was due to reduced STAT3 protein abundance this could result in inadequate LIF and IL-11 action. While LIF was not significantly reduced in the endometrial glands of women with infertility, LIF action could be impaired in women with low glandular pSTAT3. Similarly, some women had moderate to high IL-11 and low pSTAT3 in the glands. This could similarly result in abnormal IL-11 action. Further studies are required to determine the mechanisms of action of IL-11 and LIF in endometrial epithelium. In addition, LIF levels did not differ in uterine glandular epithelium between infertile and fertile women indicating IL-11 and LIF may have different roles in women with unexplained infertility.

In contrast to the present study IL-11, IL-11Rα and LIF staining were decreased in glandular epithelial cells in women with endometriosis associated infertility [12]. This suggests the endometrial phenotype of women with primary infertility differs from that of women with endometriosis associated infertility. It is likely that different therapeutic strategies may be necessary to treat endometrial implantation failure depending upon aetiology.

IL-11, pSTAT3, IL-11Rα and LIF staining was overall low and patchy or absent in luminal epithelium of both infertile and fertile women. Few tissues contained luminal epithelium making it difficult to analyze the immunostaining in this cellular compartment.

IL-11 like LIF is present in the uterine secretome indicating both could act on the blastocyst or the surface luminal epithelium. The human blastocyst expresses gp130 and LIFRα [35] but whether it also expresses IL-11Rα is not known. Although the function of IL-11 and pSTAT3 in endometrial glands needs to be elucidated, unexplained infertility in some women may be due to reduced levels of either IL-11 or pSTAT3.

## Conclusion

Importantly, our study indicates that reduced pSTAT3 and IL-11 are associated with unexplained infertility and play a role in the secretory transformation of uterine glandular epithelium during the receptivity. The factors that regulate the deficiency in IL-11 and pSTAT3 and the functional consequence of this in endometrial epithelium remain to be explored. Understanding the mechanisms of IL-11 and pSTAT3 action in endometrial epithelium may provide new therapeutic strategies for unexplained infertility.

## Competing interests

The authors declare that they have no competing interests that would prejudice the impartiality of this scientific work.

## Authors' contributions

All authors have read and approved the manuscript. ED designed the studies, performed immunohistochemistry for pSTAT3, analysed the data and drafted the manuscript. AS participated in designing the studies and collection of tissues. Y-LT performed immunohistochemistry for IL-11, IL-11Ralpha and LIF and participated in analysing the data. LAS participated in the design of the studies. JRAS participated in design of the studies, recruitment of subjects and collection of tissues.
